# The Development of Nonalcoholic Fatty Liver Disease and Metabolic Syndromes in Diet-Induced Rodent Models

**DOI:** 10.3390/life13061336

**Published:** 2023-06-07

**Authors:** Bayan Abdulhafid Aljahdali, Adnan Salem Bajaber, Doha M. Al-Nouri, Abdulrahman Saleh Al-Khalifah, Shaista Arzoo, Abeer Abdullah Alasmari

**Affiliations:** Department of Food and Nutrition Sciences, College of Food and Agriculture Sciences, King Saud University, Riyadh 1495, Saudi Arabia; 439203440@student.ksu.edu.sa (B.A.A.); abajaber@ksu.edu.sa (A.S.B.); akhalifa@ksu.edu.sa (A.S.A.-K.); 439203421@student.ksu.edu.sa (A.A.A.)

**Keywords:** non-alcoholic fatty liver disease, fat, saturated, metabolic syndrome, carbohydrate

## Abstract

Dietary macronutrients are essential for metabolic regulation and insulin function. The present study examined the effects of different high-fat diets (HFDs) and high-carbohydrate diets (HCDs) on the development of non-alcoholic fatty liver disease and metabolic syndrome indices in healthy adult male Wistar albino rats. Forty-two rats were distributed into six groups (*n* = 7), which were fed the following for 22 weeks: (1) a control diet; (2) a high-carbohydrate, low-fat diet (HCD-LFD); (3) high-saturated-fat, low-carbohydrate diet (HSF-LCD); (4) a high-monounsaturated-fat diet (HMUSF); (5) a high medium-chain fat diet (HMCF); and a (6) a high-carbohydrate, high-fiber diet (HCHF). In comparison to the control, the body weight increased in all the groups. The HSF-LCD group showed the highest levels of cholesterol, triglyceride, low-density lipoprotein, hepatic enzyme, insulin resistance, and Homeostatic Model Assessment for Insulin Resistance. A liver histology analysis of the HSF-LCD group showed macrovesicular hepatic steatosis associated with large hepatic vacuolation. Additionally, it showed marked periportal fibrosis, especially around the blood vessels and blood capillaries. The lowest levels of fasting glycemia, insulin, and HOMA-IR were observed in the HCHF group. In conclusion, these findings show that dietary saturated fat and cholesterol are principal components in the development and progression of non-alcoholic fatty liver disease in rats, while fiber showed the greatest improvement in glycemic control.

## 1. Introduction

Non-alcoholic fatty liver disease (NAFLD) has been suggested as a hepatic manifestation of metabolic syndrome (MS) since its first description in 1980 [1]. The spread of NAFLD has reached epidemic proportions, impacting about 25% of the world’s population [2,3]. The two areas with the highest incidence rates are the Middle East and South America [4]. NAFLD patients have MS in 42% of cases, diabetes in 22% of cases, hypertension in 39% of cases, obesity in 51% of cases, and hyperlipidemia in 69% of cases [4]. In 2017, the prevalence of NAFLD in Saudi Arabia was projected to be 8,451,000 people (25.7%), and the overall population with NAFLD is expected to rise to 12,534,000 cases by 2030 [5].

A threshold of triglyceride (TG) accumulation in the liver hepatocytes of more than 5% is indicative of NAFLD. TG accumulation without considerable alcohol use or other secondary causes of steatosis results in a number of histological abnormalities. This can develop into non-alcoholic steatohepatitis (NASH), fibrosis, and cirrhosis, which can progress to hepatocellular cancer [6]. NASH (Nonalcoholic Steatohepatitis) is a significant contributor to chronic liver disease and liver transplantation in many countries. This is because this disease is characterized by the presence of inflammatory infiltrates in the liver lobules, as well as the development of ballooning hepatocytes, which can worsen the condition. Therefore, NASH is a serious health concern that requires attention and effective management strategies for preventing further damage to the liver [7,8].

Insulin resistance (IR) is assumed to play a critical role in the development of the illnesses of MS, such as obesity, dyslipidemia, and hypertension. In addition, there is a pathologic transition from fatty liver to the advanced stages of NAFLD, although the exact processes behind this are still not fully understood [9,10]. Day and James initially developed the “two-hit model”, which posited that simple steatosis (the first “hit”) is necessary for the development of NASH (the second “hit”), in addition to other components, which are mostly associated with an increase in oxidative stress [11,12]. This initial theory has since been modified into a “multiple parallel-hit” model. In the presence of a significant accumulation of fat in the hepatocytes and systemic and hepatic IR, multiple concurrent alterations result in an imbalance between the liver’s antilipotoxic defense system (mitochondrial beta oxidation) and the free radical production in the gut and adipose tissue in this model. This leads to endoplasmic stress, oxidative stress, and hepatocyte apoptosis [13].

Despite the fact that the specific origin of NAFLD is unknown, dietary fat and cholesterol have been related to the development of NAFLD and NASH in both human and animal models, and a recent rise in simple carbohydrate intake has been postulated as another possible contributory factor to this [14,15]. Because of the growing recognition that diet plays a role in the pathogenesis of NAFLD, a variety of diet-induced animal models have been developed. Aside from those based on nutritional deficiencies, the majority of these models are based on a high-fat diet with varying levels of simple carbohydrates and cholesterol [16,17,18]. The particular functions of fat, carbohydrates, and cholesterol in the development of NAFLD, however, are uncertain. As a result, more studies are required to improve our understanding of the particular contributions of fat, simple carbohydrates, and cholesterol to the metabolic and inflammatory characteristics of NAFLD in people. Furthermore, it will be advantageous to build animal models of NAFLD/NASH that more appropriately mimic human pathophysiology when assessing novel NAFLD/NASH therapeutics [19,20].

Presently, lifestyle and dietary modifications are the most effective treatment options for NAFLD. Weight loss and increased physical activity are two examples of these lifestyle modifications [21,22]. Losing weight and increased physical activity can minimize hepatic inflammation and hepatocellular damage, while also improving steatosis, dyslipidemia, IR, and cardiovascular risk. Furthermore, all NAFLD patients are advised to follow a number of dietary restrictions, including avoiding simple carbohydrates, saturated fats, and sweetened drinks, as well as consuming a diet rich in fruits and vegetables [23,24]. The purpose of this study was to evaluate how high-fat, high-fiber, and high-carbohydrate diets affect the development of NAFLD and MS in Wistar albino rats. As a symptom of hepatic steatosis, NAFLD advancement was investigated histologically and biochemically over time to analyze the disease’s progression.

## 2. Materials and Methods

### 2.1. Animals

Forty-two healthy adult male Wistar albino rats (160–200 g, 5–6 weeks old) were obtained and maintained in the Experimental Animal Care Center at King Saud University, Riyadh, Saudi Arabia. The rats were kept in controlled, ambient conditions (22 ± 2 °C, 50% humidity, and 12/12 h light/dark) and were allowed to access their designated diets and drinking water freely. At the end of the first week, the diets of all the groups, except the control group, were changed to five different experimental diets for 22 weeks. 

### 2.2. Diet

The 42 rats were distributed into six groups and group housed (*n* = 7/cage) as follows. Group 1 (control) received a standard diet with 15% of their calories coming from fat (Cat. No. D12450H, Research Diets, New Brunswick, NJ, USA). Group 2 (HCD-LFD) received a high-carbohydrate, low-fat diet with 60% kcal coming from sucrose. Group 3 (HSF-LCD) received a high-saturated-fat, low-carbohydrate diet with 45% kcal coming from animal fat (butter). Group 4 (HMUSF) received a high-monounsaturated-fat diet with 45% kcal coming from Spanish organic extra virgin olive oil (first extraction). Group 5 (HMCF) received a high medium-chain fat diet with 45% kcal coming from organic extra virgin coconut oil. Group 6 (HCHF) received a high-carbohydrate, high-fiber diet with 64% kcal coming from oat bran. The diet components were prepared daily by mixing the components well to create a dough-like consistency. The diet was then shaped into pellets and kept on a large heat pad with a fan until thoroughly dried to create a more solid form of food. They were kept segregated at 4 °C to avoid fungus contamination (Table 1).

### 2.3. Assessment of Body Weight (BW) and Food Consumption

The rats’ BWs were recorded weekly throughout the experimental period and their food intake was recorded daily. BW was recorded in the non-fed state at the beginning of the study (initial weight) and at the time before euthanization (final weight) to calculate the weight gain and growth rate:

Weight gain = Final BW (g) − Initial BW (g)

Growth rate = Total weight gain (g)/duration

Food consumption was measured daily in all the experimental groups by calculating the difference between the diets provided (before consumption) and the diet unconsumed, using a calibrated scale with 0.01 mg precision:

Food consumption per day/group (g) = diet provided (g) − diet unconsumed (g)

Food efficiency (FE) was calculated as follows:

Food efficiency = (weight gain)/(total food consumption)

### 2.4. Rat Euthanization and Sample Collection

At the end of the 22 weeks, the rats were fasted for 12 h and anesthetized with intraperitoneal pentobarbital sodium (60 mg/kg BW) [25]. A plain tube was used to obtain blood samples using a direct cardiac puncture, which were centrifuged at a speed of 3000 rpm for 10 min to collect the serum, followed by storage at −20 °C for further biochemical analyses.

### 2.5. Liver Histology

The samples for the histopathological examination were obtained from the caudate and middle lobes of the livers in the different groups and then fixed in 10% neutral buffered formalin. The tissues were dehydrated, cleared, and embedded in paraffin, to be then cut into 5 μm thick sections. Hematoxylin stained the nuclei of the cells blue and eosin stained the cytoplasms with a pink color. The scoring of hepatic steatosis was performed according to Nassir et al. [26], in which grading was performed on the basis of the percentage of fat within the hepatocytes: grade 0 (healthy, <5%), grade 1 (mild, 5–33%), grade 2 (moderate, 34–66%), and grade 3 (severe, >66%). Sudan Black B staining, to stain the lipids with varying degrees of black-colored pigments, is seen in the positive reaction, and hematoxylin and eosin (H and E) staining were performed on serial sections [27]. For an assessment of the fibrosis extent within the hepatic tissues, Masson’s trichrome staining was performed [28]. 

### 2.6. Biochemical Analysis

The parameters measured included the serum levels of total cholesterol (TC), low-density lipoprotein cholesterol (LDL-C), high-density lipoprotein cholesterol (HDL-C), and triglycerides (TGs), which were determined using specific rat assay kits (Cat. No. MBS168179, MBS8806454, MBS8804648, and MBS2700533, Mybiosource, San Diego, CA, USA, respectively). The glucose and insulin were measured using rat ELISA kits (Cat No. MBS7233226, MBS28912, Mybiosource, San Diego, CA, USA, respectively). The IR was estimated according to the Homeostasis Model Assessment (HOMA-IR), which was calculated using the following [29]:

$$\mathrm{HOMA}-\mathrm{IR}=\frac{glucose\left(\frac{mg}{dL}\right)\times insulin(\frac{\mu U}{mL})}{405}$$

The serum alanine aminotransferase (ALT) and serum aspartate aminotransferase (AST) were measured using ALT and AST ELISA kits (Cat No. MBS8801684 and MBS8804612, Mybiosource, San Diego, CA, USA, respectively).

### 2.7. Statistical Analysis

The data were analyzed using SPSS^®^ software version 24.0. (SPSS Inc., Chicago, IL, USA). The normality was tested using the Kolmogorov–Smirnov test. Descriptive statistics were used to compute the means and standard deviations in order to examine the characteristics of the study groups. A one-way analysis of variance (ANOVA) and then Tukey’s *t*-test were used to identify the differences in the study groups according to the study variables, and *p*-values less than 0.05 were considered as statistically significant. A Kruskal–Wallis test was used in the H and E scoring.

## 3. Results

### 3.1. Effect of Diets on BW and Food Consumption

**Figure 1:**
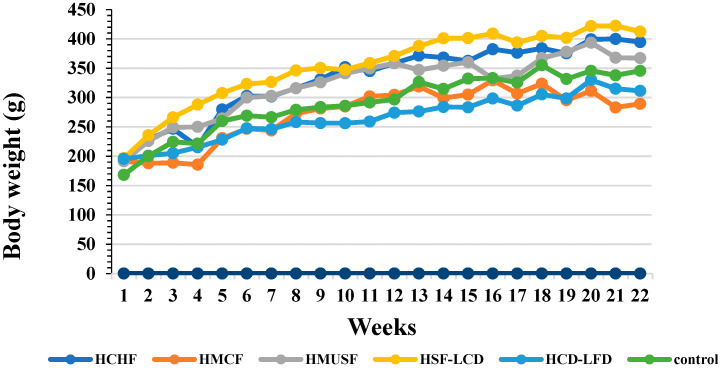
Effect of high-carbohydrate, high-fat, and high-fiber diets consumption on body weight during 22 weeks.

**Figure 2:**
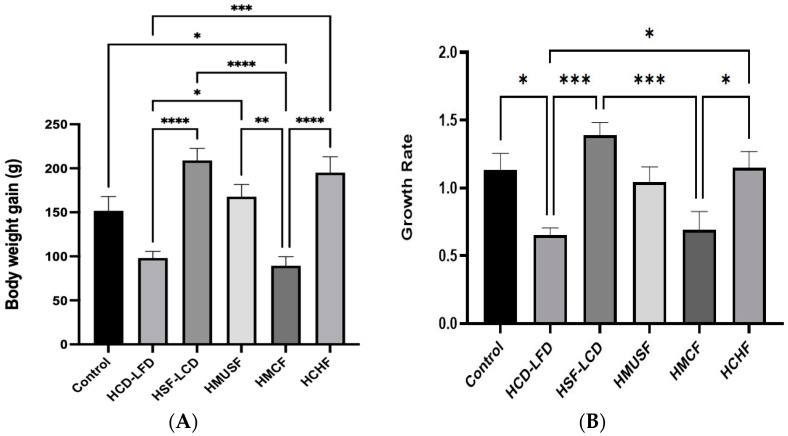

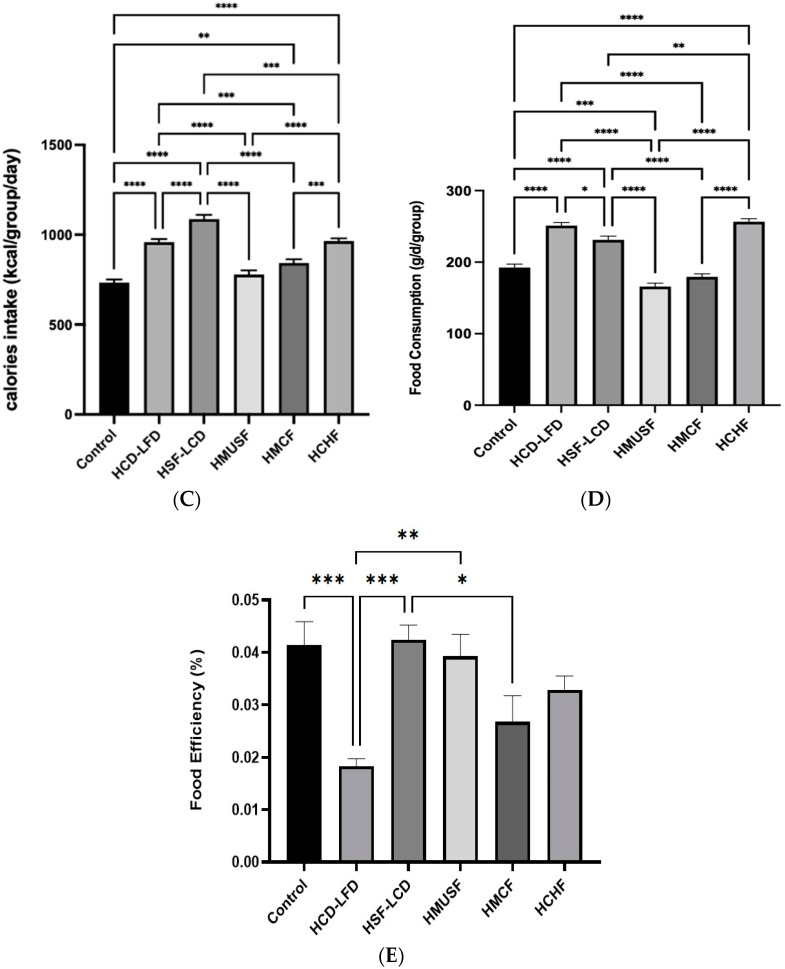
Effect of high-carbohydrate, high-fat, and high-fiber diets consumption on (**A**) BW gain, (**B**) growth rate, (**C**) calories intake, (**D**) food consumption, and (**E**) food efficiency. Data are presented as means ± SD, *n* = 7/gp. * *p*-value < 0.05; ** *p*-value < 0.005; *** *p*-value < 0.0005; and **** *p*-value < 0.0001. HCD-LFD—high-carbohydrate, low-fat diet; HSF-LCD—high-saturated-fat, low-carbohydrate diet; HMUSF—high-monounsaturated-fat diet; HMCF—high medium-chain fat diet; and HCHF—high-carbohydrate, high-fiber diet.

Initially, all six study groups had comparable BWs. After the experiment, the highest BW was observed in the HSF-LCD group, and the lowest one was observed in the HMCF group (Figure 1). A statistically significant difference in the BW gain between the control and HMCF group was observed. Among the experimental groups, statistically significant differences were observed between the HSF-LCD, HCD-LFD, and HMCF groups and between the HMCF, HMUSF, and HCHF groups (Figure 2A).

The growth rate for the HSF-LCD group was the highest, while the lowest was observed in the HCD-LFD group. A statistically significant difference in the growth rate (*p* < 0.05) between the control and HCD-LFD experimental group was observed. Among the experimental groups, statistically insignificant differences were observed between the HCD-LFD, HMUSF, and HMCF groups and between the HSF-LCD, HMUSF, and HCHF groups for growth rate (Figure 2B).

The calorie intake for the HSF-LCD group was the highest, while the lowest was observed in the control group. Statistically significant differences in calorie intake were observed between the control and all the experimental groups expect the HMUSF group. Among the experimental groups, a statistically insignificant difference was observed between the HMUSF and HMCF groups and between the HCD-LFD and HCHF groups (Figure 2C).

The food consumption for the HCHF group was the highest, followed by the HCD-LFD, HSF-LCD, control, HMUSF, and HMCF groups. Statistically significant differences for food consumption were observed between the control and experimental groups except the HMCF group. Among the experimental groups, a statistically insignificant difference was observed between the HMUSF and HMCF groups (Figure 2D). The highest food efficiency (FE) was noted in the HSF-LCD group, while the lowest was observed in the HCD-LFD group. Statistically insignificant differences for FE were found between the control and other groups (except HCD-LFD) (Figure 2E).

### 3.2. Effect of Diets on Lipid Profile

**Figure 3:**
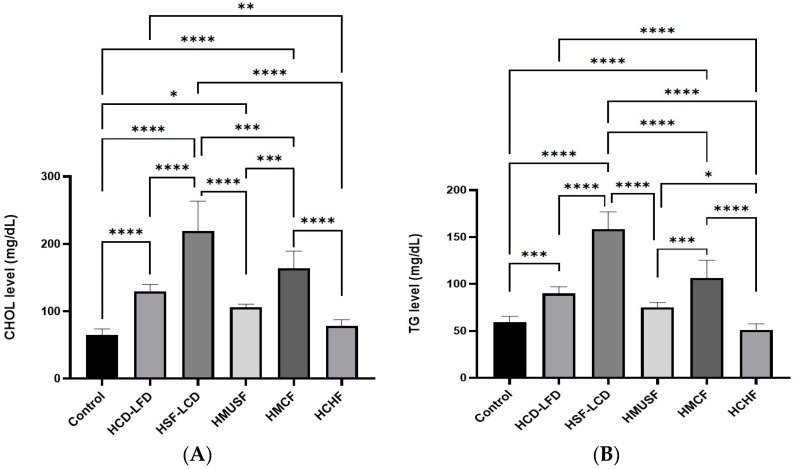

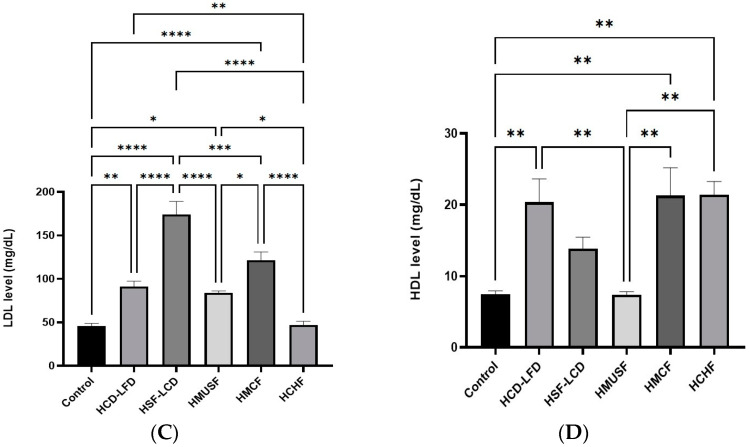
Effect of high-carbohydrate, high-fat, and high-fiber diets consumption on (**A**) cholesterol (CHOL), (**B**) triglycerides (TG), (**C**) low-density lipoprotein (LDL) levels, and (**D**) high-density lipoprotein (HDL), (mg/dL). Data are presented as means ± SD, *n* = 7/gp. * *p*-value < 0.05; ** *p*-value < 0.005; *** *p*-value < 0.0005; and **** *p*-value < 0.0001. HCD-LFD—high-carbohydrate, low-fat diet; HSF-LCD—high-saturated-fat, low-carbohydrate diet; HMUSF—high-monounsaturated-fat diet; HMCF—high medium-chain fat diet; HCHF—high-carbohydrate, high-fiber diet.

Figure 3 shows the effect of the experimental diets on the lipid profiles. The highest levels of cholesterol (Figure 3A), TG (Figure 3B), and LDL (Figure 3C) were observed in the HSF-LCD group, while the lowest levels were observed in the HCHF group. The control group and experimental groups showed statistically significant differences in their cholesterol and LDL levels, except for the HCHF group. For the TG level, the control group and experimental groups showed statistically significant differences, except for the HMUSF and HCHF groups. There were statistically significant differences between the HSF-LCD group and other groups (HCD-LFD, HMUSF, HMCF, and HCHF) in their cholesterol, TG, and LDL Levels. The HDL level (Figure 3D) was highest in the HCHF group and lowest in the HMUSF group compared with the reference range (7.44 ± 1.23 mg/dL). The control group and other groups (HCD-LFD, HMCF, and HCHF) showed statistically significant differences in their HDL levels. 

### 3.3. Effect of Diets on Glycemic Control

Table 2 illustrates the experimental diets’ effect on the parameters of glycemic control, such as the glucose, insulin, and HOMA-IR levels. These levels were highest in the HSF-LCD group and lowest in the HCHF group. The results showed statistically significant differences between the HSF-LCD group and all other experimental groups for their insulin, glucose and HMOA-IR levels. The results showed statistically significant differences between the HCHF, HCD-LFD, HSF-LCD, and HMCF groups and also between the HMUSF, HCD-LFD, HSF-LCD, and HMCF groups for their insulin levels. In addition, the results showed statistically significant differences between the HCHF, HSF-LCD, and HMCF groups for their glucose levels and the HCHF, HCD-LFD, HSF-LCD, and HMCF groups for their HOMA-IR levels.

### 3.4. Effect of Diets on Liver Enzymes

**Figure 4:**
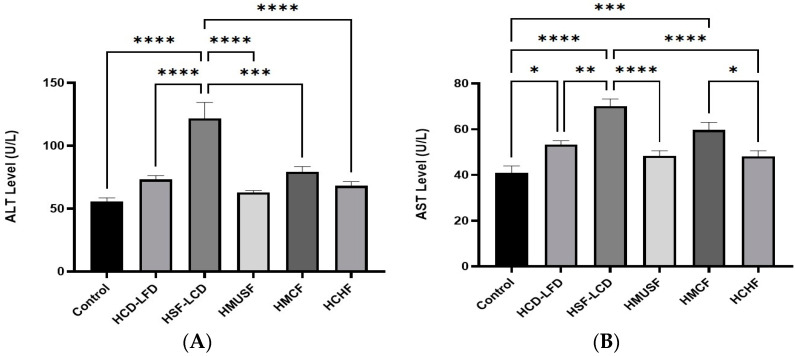
Effect of high-carbohydrate, high-fat, and high-fiber diets consumption on (**A**) alanine aminotransferase (ALT) and (**B**) aspartate aminotransferase (AST). Data are presented as means ± SD, *n* = 7/gp. * *p*-value < 0.05; ** *p*-value < 0.005; *** *p*-value < 0.0005; and **** *p*-value < 0.0001. HCD-LFD—high-carbohydrate, low-fat diet; HSF-LCD—high-saturated-fat, low-carbohydrate diet; HMUSF—high-monounsaturated-fat diet; HMCF—high medium-chain fat diet; HCHF—high-carbohydrate, high-fiber diet.

Figure 4 demonstrates the effect of the experimental diets on liver enzymes. The highest levels of ALT (Figure 4A) and AST (Figure 4B) were observed in the HSF-LCD group, while the lowest levels of ALT and AST were in the HMUSF and HCHF groups, respectively. A statistically significant difference was observed in both the enzyme levels between the control and some experimental groups (*p* < 0.05). The results show an insignificant difference between the HMUSF and control groups for both their ALT and AST levels and statistically significant differences between the HSF-LCD, HCD-LFD, HMUSF, HMCF, and HCHF groups for their ALT levels. Additionally, there were statistically significant differences between the HSF-LCD, HCD-LFD, HMUSF, and HCHF groups in their AST levels.

### 3.5. Histopathological Analysis

**Figure 5:**
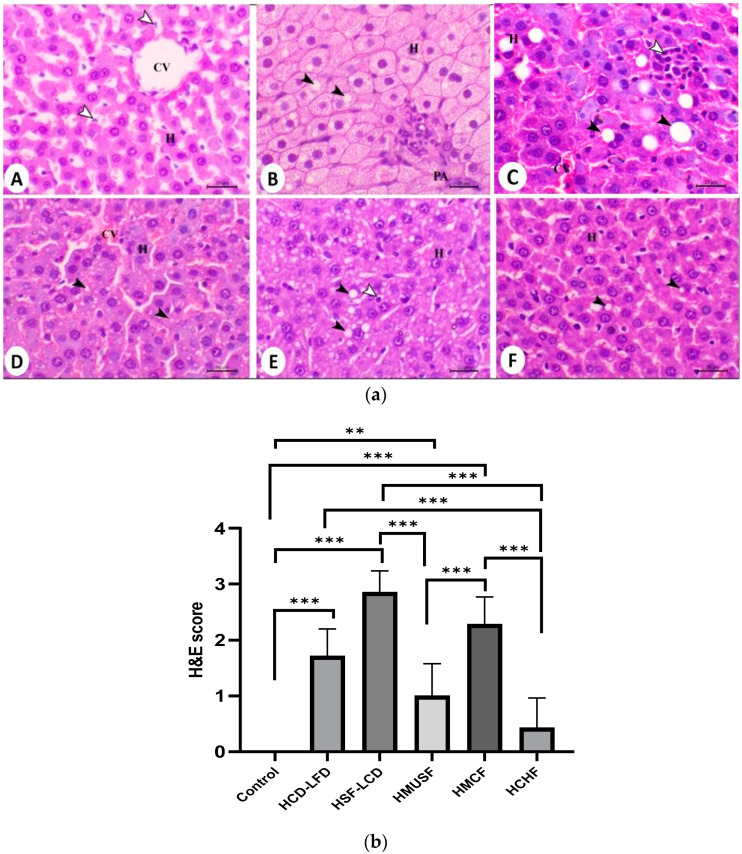
(**a**) Histological evaluation of liver sections from all groups of rats representative H and E stains ((**H**,**E**), ×400, bar = 20 µm). (**A**)—Control; (**B**)—HCD-LFD (high-carbohydrate, low-fat diet); (**C**)—HSF-LCD (high-saturated-fat, low-carbohydrate diet); (**D**)—HMUSF (high-monounsaturated-fat diet); (**E**)—HMCF (high medium-chain fat diet); (**F**)—HCHF (high-carbohydrate, high-fiber diet). (**b**) Scoring of hepatic steatosis by H and E stains from experimental groups. Data are presented as means ± SD, *n* = 7/gp. ** *p*-value < 0.001 and *** *p*-value < 0.0001. HCD-LFD—high-carbohydrate, low-fat diet; HSF-LCD—high-saturated-fat, low-carbohydrate diet; HMUSF—high-monounsaturated-fat diet; HMCF—high medium-chain fat diet; HCHF—high-carbohydrate, high-fiber diet.

Figure 5a,b show the histology examination results. Hepatic steatosis was observed in the HSF-LCD group. A statistically significant difference in the H and E score between the control and all the experimental groups was observed, except for the HCHF group. Among the experimental groups, statistically significant differences were found between the HSF-LCD, HMUSF, and HCHF groups and between the HCD-LFD and HCHF groups. The livers of the control rats showed no pathological alterations. The control group (A) showed normal hepatocytes arranged in cords and separated with blood sinusoids around the central vein (H indicates hepatocytes, CV indicates central vein, and arrows indicate sinusoidal Kupffer cells), which placed them in grade 0. The HCD-LFD group (B) showed marked cell swelling associated with excessive glycogen storage and microvesicular steatosis (black arrows) (PA indicates portal area), which were in the score range of grade 2. The HSF-LCD group (C), which was found to be in grade 3, showed marked macrovesicular hepatic steatosis associated with large hepatic vacuolation (black arrows) and foci of lipogranuloma accompanied by focal hepatic necrosis, which was associated with mononuclear inflammatory cell infiltration that mostly consisted of macrophages (white arrow). The HMUSF group (D) showed mild fatty changes within their hepatic cells (black arrows), which placed them in grade 1. The HMCF group (E) showed a moderate decrease in fatty changes within their hepatic cells (black arrows) and hypertrophy of their Kupffer cells (white arrow), which denoted them to be in grade 2. The HCHF group (F), which was found to be in grade 0, showed a marked decrease in fatty changes within their hepatic cells (black arrows indicate few fat droplets within the cytoplasm of hepatocytes).

**Figure 6:**
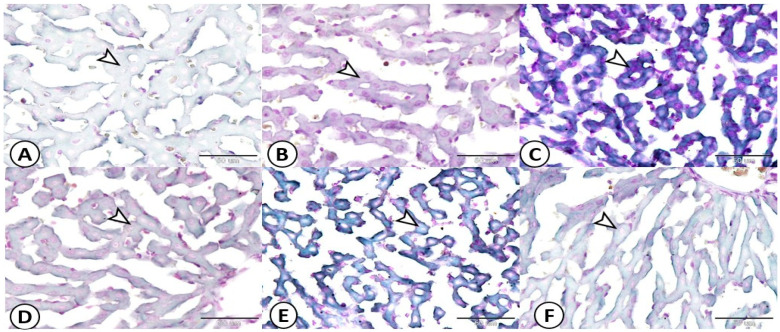
Photomicrograph of hepatic tissues of different treated groups. Sudan black B, ×200, bar = 50 µm. (**A**)—Control; (**B**)—HCD-LFD (high-carbohydrate, low-fat diet); (**C**)—HSF-LCD (high-saturated-fat, low-carbohydrate diet); (**D**)—HMUSF (high-monounsaturated-fat diet); (**E**)—HMCF (high medium-chain fat diet); (**F**)—HCHF (high-carbohydrate, high-fiber diet).

Figure 6 shows a photomicrograph of the hepatic tissues of different groups. The livers from the control group (A) showed a scant amount of lipid in the cytoplasms of their hepatocytes (arrow). The livers of the HCD-LFD and HMCF groups (B, E) showed moderate amount of lipid in cytoplasms of their hepatocytes (arrow). The livers from the HSF-LCD group (C) showed a high amount of lipid in the cytoplasms of their hepatocytes (arrow). The livers from the HMUSF group (D) showed a slight amount of lipid in the cytoplasms of their hepatocytes (arrow). The livers from the HCHF group (F) showed a mild amount of lipid within the cytoplasms of their hepatocytes (arrow) (Sudan black B stain bar = 50 µm).

**Figure 7:**
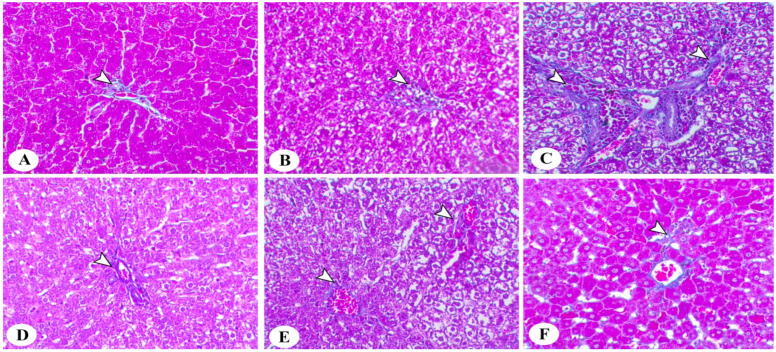
Photomicrograph of hepatic tissues of different treated groups. Masson’s trichrome stain B, ×200, bar = 50 µm. (**A**)—Control; (**B**)—HCD-LFD (high-carbohydrate, low-fat diet); (**C**)—HSF-LCD (high-saturated-fat, low-carbohydrate diet); (**D**)—HMUSF (high-monounsaturated-fat diet); (**E**)—HMCF (high medium-chain fat diet); (**F**)—HCHF (high-carbohydrate, high-fiber diet).

Figure 7 shows a photomicrograph of the hepatic tissues of different groups stained with Masson’s trichrome stain. The control group (A) showed a mild periportal circular layer of fibrous connective tissues. The HCD-LFD group (B) showed a mild increase in this fibrous layer. The HSF-LCD group (C) showed marked periportal fibrosis, especially around their blood vessels and blood capillaries. The HMUSF group (D) showed a mild increase in the fibrous layer within the periportal area. The HMCF group (E) showed an increase in periportal fibrous connective tissues. The HCHF group (F) showed an increase in fibrous layer connective tissues. (arrowheads demonstrate the fibrous connective tissues).

## 4. Discussion

Sedentary behavior and consuming large amounts of high-trans-fat foods have been important contributors to the increasing obesity pandemic and its related comorbidities. One of these results is NAFLD. However, the pathophysiology of this widespread illness is still unknown. Furthermore, the function of increased trans fat consumption in NASH development has not been extensively studied [30]. Most studies have focused primarily on the effects of high-fat diets (HFDs) on metabolism, but complex carbohydrates and different types of fat have received little attention. This study combined the primary dietary and behavioral components reported to contribute to MS and its comorbidities to produce an NAFLD rodent model that closely reflects diet and lifestyle. This study examined two high-carbohydrate diets’ (HCDs) and three HFDs’ effects on metabolic regulation, IR, and NAFLD in normal Wistar rats.

NAFLD is often associated with obesity. Milić et al. [31] showed that 80% of patients with NAFLD were obese (body mass index (BMI) > 30 kg/m^2^). The large amount of visceral adipose tissue (VAT) in morbidly obese individuals (BMI > 40 kg/m^2^) leads to a high morbidity resulting from NAFLD. Under normal situations, the liver does not store TAG. Stressful situations, such as obesity, can cause a hepatic lipid buildup. High-fat or high-carbohydrate diets lead to abnormal lipid metabolism that results in ectopic hepatic lipid buildups [26]. Obesity is linked to an increase in the glucocorticoid stress hormone cortisol, which plays a role in the development of obesity. In addition to increasing appetite and causing a preference for energy-dense food, glucocorticoids can also redistribute white adipose tissue to the abdominal region and suppress brown adipose tissue activity, resulting in abdominal obesity and its metabolic complications [32]. Furthermore, NASH patients consumed 14% of their total energy from saturated fatty acid, compared to 10% among controls, according to Musso et al. [33]. In addition, glutathione is a cofactor for numerous enzymes that impact the responses associated with obesity. Saturated fat intake is associated with an impaired glutathione metabolism, which leads to oxidative stress and NAFLD progression [34]. Another study conducted by Jensen et al. [19] on rats found that rats fed with a high-fat diet became obese at week 8 [19]. 

Obesity and fat on the liver also affect ALT and AST levels. Several studies involving morbidly obese people undergoing bariatric surgery and patients at hepatology clinics undergoing liver biopsies have discovered that the ALT levels were higher in the presence of NASH than in those with steatosis in the early stages [35]. These metabolic changes were more closely reflected in the HSF-LCD group, which showed the highest BW gain among the groups. According to an additional study, obesity could raise the DNA methylation in liver tissue via boosting oxidative stress, which ultimately results in a degeneration of liver tissue. Additionally, adipokines, resistin, leptin, visfatin, and tumor necrosis factor α (TNF-α) are proteins secreted by visceral adipose tissues that can affect how the liver functions and cause inflammation, cirrhosis, and hepatocellular cancer [36]. However, in this study, we did not test these parameters; therefore, further studies need to address the possible mechanisms of TNF-α and adipokines on NAFLD progression.

The difference in the calorie intake between the HFD and HCD groups may have been due to the HFDs having a higher satiating effect due to their slower digestion in comparison to the HCDs, which led to the rats being more efficient with their ingested calories [37,38]. Furthermore, the HCD group had a lower calorie density than the HFD group, which could explain some of these findings. Most digestible carbs have 4 kcal of accessible energy per gram, whereas fat provides 9 kcal per gram [39]. However, since the diets were prepared differently, the control diet was given in the form of pellets from Research Diets, while all the other diets were freshly made daily as pellets [40]. As a result, changes in the meal consistencies may have resulted in variances in their food consumption compared to that of the control group. In addition, we noticed that, during the experiment, the HMCF group began to eat less. This may have been due to the so-called intestinal permeability “Leaky gut.” Some types of short- and medium-chain fats and types of carbohydrates have been associated with a change in the gut microbiota’s metabolites, which can cause a leaky gut and have an impact on intestinal and systemic health issues. However, more investigation is required to understand the effect of the length of the fatty chain on the gut microbiome [41].

The proportion of fat in the liver indicates the balance between the FFA flow via lipolysis, fatty acid oxidation, de novo lipogenesis, and VLDL secretion [42]. Therefore, increased circulating TG and LDL-C levels and lower HDL-C levels are characteristic of dyslipidemia associated with NAFLD [43]. Only the rats in the HSF-LCD group developed dyslipidemia, which was characterized as hypertriglyceridemia. The development of hepatic steatosis in the HFD-fed rats was consistent with previous findings in other rodent species [14], demonstrating that fat and cholesterol are significant factors in the development of experimental NAFLD/NASH. The addition of dietary cholesterol (NASH diet) resulted in the most harmful liver abnormalities, as evidenced by a large hepatic TG and cholesterol buildup, noticeable declines in the liver density, and severe morphological changes in the liver histology [44].

Polyunsaturated fatty acids, such as α-linolenic acid (ω-3) and linoleic acid (ω-6), have been linked to NAFLD. ω-6 PUFAs are mainly composed of linoleic acid (LA), which is metabolized to arachidonic acid, and ω-3 PUFAs are mainly composed of α-linolenic acid (ALA), which is metabolized to eicosapentaenoic acid (EPA) and docosahexaenoic acid (DHA) [45,46]. Arachidonic acid is proinflammatory, prothrombotic, and proaggregatory, while eicosapentanoic acid (EPA) and docosahexanoic acid (DHA) modulate the liver’s lipid composition, increasing its anti-inflammatory mediators and decreasing IR [47]. The balance between ω-3 and ω-6 PUFAs is important in human health and the optimum ratio (ω-6/ω-3) in the diet is four to one. However, studies have shown that most NAFLD patients have a higher level of ω-6 and a lower level of ω-3 PUFAs [45,46], and this ratio can be 20–25: 1 [45]. As a result, the ratio of ω-6 to ω-3 plays a significant role in increasing the prevalence of chronic metabolic diseases [48].

The diet of the HSF-LCD group was based on saturated fats, which are linked to the progression of NAFLD pathophysiology. The ratio of the polyunsaturated/saturated fatty acid consumption in NASH patients was recently discovered to be lower than that in the healthy population [49]. Intestinal incretin glucose-dependent insulinotropic polypeptide (GIP) appears to play a key role in the link between saturated fats and NASH. The GIP levels are elevated for a longer time in NASH patients after consuming saturated fat, and this enhanced GIP response is linked to the severity of liver disease [50]. Additionally, a higher saturated fat intake correlates with an impaired glutathione metabolism towards oxidative stress, leading to NAFLD progression [51]. 

In humans, NAFLD is commonly related to IR and obesity. IR increases de novo lipogenesis, which increases the possibility of NAFLD and indirectly increases the free fatty acid flow to the liver by lowering the inhibition of lipolysis [52]. These metabolic abnormalities were more apparent in the HSF-LCD group. Accordingly, the HSF-LCD-fed rats became obese and had higher glucose, insulin, and HOMA-IR levels, indicating disturbances in their glucose metabolism and insulin fasting levels. At the same time, the HMUSF and HCHF groups had the best diets for improving the glycemic index. 

Significant increases in both hepatic TG and cholesterol, considerable losses in liver density, and severe morphological modifications in the histology in the HSF-LCD group were accompanied by higher plasma levels of ALT and AST, indicating decreased liver function. These findings were consistent with recent findings in animals [41,48] and humans [49], which indicate that combining dietary fat and carbohydrates or fat and cholesterol [50] might cause faster and more severe effects on the liver. In another study on humans, however, statistically insignificant associations were found between a fatty diet and liver enzymes [51]. 

Xu et al. [52] investigated the effects of HFDs on some parameters. They found that HFDs increased the glucose, TG, cholesterol, ALT, FFAs, insulin, and TNF-α levels in rats, leading to increased BWs. In addition, steatohepatitis developed, which appeared in the H and E staining and was used to study the histology of the liver [52]. At the same time, in the HSF-LCD group in our study, the liver histology via H and E staining showed marked hepatic steatosis associated with large hepatic vacuolation and the hypertrophy of Kupffer cells. As for fibrosis, the same group—the HSF-LCD group—showed marked periportal fibrosis, especially around their blood vessels and blood capillaries. This was consistent with the hypothesis that fats affect the liver tissue and may contribute to fibrosis after a period of high-fat consumption.

Medium-chain fatty acids are recognized for being easily absorbed and processed in the body. Medium-chain fatty acids, such as virgin coconut oil, have been proven to promote the beta-oxidation of fat, thus protecting against non-alcoholic fatty liver [53]. According to a recent study, virgin coconut oil consumption was linked to metabolic dysfunctions, adipose inflammation, and hepatic lipid buildup, as well as higher levels of cholesterol and triglycerides [54]. In addition, coconut oil appears to promote IR over time, but does not appear to improve long-term glycemic management [55]. In another trial, consuming coconut oil affected the antioxidant activity and inflammation in adipose tissue and a significant accumulation of hepatic fat and serum lipid profile. However, the ideal coconut oil dose was not determined by the authors [56]. This was reflected in the lipid profiles and IR levels observed in the HMCF group, which confirmed our findings. However, the findings in relation to this impact are still unclear.

Coconut oil is high in saturated fats, which may contribute to the development of NAFLD. Human studies have resulted in a variety of findings. According to one published study, 4 weeks of coconut oil use did not significantly alter the liver enzymes of healthy persons. However, a different study found that patients with NAFLD who used virgin coconut oil for 12 weeks saw an improvement in their liver enzyme levels [54]. In our study, this group had elevated liver enzyme levels. More investigation is required to understand the potential benefits and risks fully.

The primary indicators of liver dysfunction are elevated levels of liver enzymes. The important markers of NAFLD in the general population are liver enzymes. The Mediterranean diet is generally seen as being dependent on olive oil, one of the monounsaturated fats that can help people suffering from NAFLD liver enzymes, which could be two to four times higher than normal levels. [57]. The presence of olive oil in the HMUSF diet may explain some of these potential benefits. Olive oil includes tocopherols, carotenes, and other phenolic compounds that have antioxidant effects, in addition to monounsaturated and polyunsaturated fatty acids [58]. Olive oil’s potential mechanisms of action include improved insulin-receptor union, cellular permeability, and signaling; enhanced stomach emptying; a reduced glucose absorption; and a higher insulin sensitivity [59,60]. A supplementation of olive oil improved the glucose homeostasis in rats and elevated the glucagon-like peptide-1 (GLP-1) levels [61]. Garg et al. [62] showed that a high-monounsaturated-fat diet reduced plasma glycemia in type 2 diabetics compared to an HCD [62]. Olive oil’s beneficial effects on glucose homeostasis include enhanced GLP-1 secretion [63]. Additionally, patients with NAFLD who consumed 20 g/day for 12 weeks in a hypocaloric diet reduced the severity of their fatty liver [64]. It is worth noting that many studies have shown the effect of olive oil on lipid profiles and improvements in good cholesterol levels [65]. The results of the HMUSF group indicated a positive effect on the lipid levels, as these were lower than those in the other HFDs groups, which indicated the good effect of olive oil on lipid profiles, glycemic control, and liver enzymes. However, we noticed a slight increase in the lipid profile (LDL and TG). This may be due to the different quality, type, and processing methods, and all these factors affect the active ingredients in olive oil. More studies should be performed to determine the upper limit of consumption for fatty liver patients in terms of positive effects. These findings do not alter the current dietary recommendations and benefits of olive oil intake in general, but there is a need for greater research to clarify the complex connections between various dietary lipids and health.

In rats, dietary fiber decreases obesity and hepatic steatosis independently of fermentability [66]. A 12-week oligofructose supplementation reduced the BW and improved the glucose and lipid metabolism in humans [67]. Additionally, it was shown that oats can improve lipid profiles, liver functions, and liver enzymes, especially ALT, and reduce obesity [68]. In a double-blind randomized study, 75 hypercholesterolemic humans were randomly allocated to one of two treatments: oat or dextrose consumption. The results indicated that oats reduced TC and LDL-C considerably after the intervention, with LDL-C reductions much higher than those in the control group that ate dextrose, demonstrating that oats improved the lipid profile [69]. According to a recent study, oat beta-glucan consumption may significantly decrease the concentrations of TC and LDL [70,71]. Additionally, beta-glucan in oats could be a strategy for decreasing the lipid profile risks by improving the HDL and Apo B levels to reduce the risks of LDL [72]. These studies support our findings in the HCHF group.

Fiber deficiency is frequent in patients with NAFLD [73,74,75] and NASH [33]. The mechanism by which inadequate fiber intake may contribute to NAFLD is unknown [76]. β-glucan fiber, which is found in oats, has been related to good gut flora. Human microbiotas are influenced by prebiotics via lowering luminal pH and preventing pathogen growth [76,77,78]. Inflammation caused by bacterial translocation into the systemic circulation increases IR and damage to the liver [79]. Therefore, a therapeutic change in intestinal bacterial flora has been proposed for the treatment of NASH [76]. Additionally, a series of studies have found that oats or β-glucan have favorable metabolic benefits for patients with and without type 2 diabetes [80,81,82]. An oat-based diet containing β-glucan improves the metabolic and anthropometric profiles in type 2 diabetics, showing large decreases in glycosylated hemoglobin A1c (HbA1c), weight, and BMI, as well as high HDL cholesterol levels [83].

When a surplus dosage of barley β-glucan supplement (6.31 g β-glucan) is provided in an HCD, it improves the glucose and insulin responses [82]. Oat fiber reduces the glucose intolerance and insulin sensitivity in individuals with MS [84]. Additionally, β-glucan reduces fat storage by raising energy expenditure through a process involving changes in the composition of the hepatic bile acids [85]. In the HCHF group, the glycemic control levels were close to those of the control group, but better than those of the other experimental groups.

## 5. Conclusions

Despite their varied food consumption, all the groups had increased BWs, to varying degrees, after the 22-week treatment. The consumption of oats in the HCHF group and olive oil in the HMUSF group, compared to other HFDs or HCDs, had the most positive impacts on glycemic management and benefits in lowering the blood and hepatic lipids. These findings demonstrate the intricate interrelationship between glucose and lipid metabolism, since it is difficult to change one without affecting the other. Furthermore, not only is fat composition significant because of its negative impact on glycemic and lipidic regulation, but the type of fat also influences metabolic control and can stimulate NAFLD. Additionally, the results of our study can contribute to the interest in eating beneficial fats and fiber in the diet programs or dietary habits of NAFLD patients, because of their healthy effects on the liver and ability to delay the progression of the disease. Still, there is a need for more research to determine their appropriate doses. In order to establish innovative techniques for the prevention and treatment of this widespread liver illness, further research is needed to define the biochemical processes and molecular pathways generated by the dietary habits that are involved in the development and progression of NAFLD/NASH.

## Figures and Tables

**Table 1:** Diet composition and energy intake for control and experimental diets.

	Group 1 Control	Group 2 HCD-LFD	Group 3 HSF-LCD	Group 4 HMUSF	Group 5 HMCF	Group 6 HCHF
Protein	20%	20%	20%	20%	20%	20%
Carbohydrate	65%		35%	35%	35%	
Sucrose		60%				
Oat						64%
Fat	15%	20%				16%
Animal fat			45%			
Olive oil				45%		
Coconut oil					45%	
Vitamin & Mineral mix	0.30%	0.30%	0.30%	0.30%	0.30%	0.30%
Energy density	3.81 Kcal/g	3.82 Kcal/g	4.7 Kcal/g	4.7 Kcal/g	4.7 Kcal/g	3.76 Kcal/g

HCD-LFD—high-carbohydrate, low-fat diet; HSF-LCD—high-saturated-fat, low-carbohydrate diet; HMUSF—high-monounsaturated-fat diet; HMCF—high medium-chain fat diet; HCHF—high-carbohydrate, high-fiber diet.

**Table 2:** Effect of high-carbohydrate, high-fat, and high-fiber diets consumption on insulin, glucose, and HOMA-IR.

Groups	Control	HCD-LFD	HSF-LCD	HMUSF	HMCF	HCHF
Glucose (mg/dL)	73.86 ± 5.18 ^c,e^	93.57 ± 5.19 ^c^	181.00 ± 35.67 ^a,b,d,e,f^	86.33 ± 4.55 ^c^	100.57 ± 13.35 ^a,c,f^	72.86 ± 6.87 ^c,e^
Insulin (mIU/mL)	0.08 ± 0.03 ^b,c,e^	0.72 ± 0.12 ^a,c,d,e,f^	1.96 ± 0.19 ^a,b,d,e,f^	0.14 ± 0.02 ^b,c,e^	1.38 ± 0.13 ^a,b,c,d,f^	0.08 ± 0.04 ^b,c,e^
HOMA-IR	0.0150 ± 0.0062 ^b,c,e^	0.1666 ± 0.0241 ^a,c,e,f^	0.8774 ± 0.2019 ^a,b,d,e,f^	0.0303 ± 0.0039 ^c,e^	0.3417 ± 0.0530 ^a,b,c,d,f^	0.0140 ± 0.0071 ^b,c,e^

Data are presented as means ± SD, *n* = 7/gp. HCD-LFD—high-carbohydrate, low-fat diet; HSF-LCD—high-saturated-fat, low-carbohydrate diet; HMUSF—high-monounsaturated-fat diet; HMCF—high medium-chain fat diet; HCHF—high-carbohydrate, high-fiber diet. The values with different superscript letters in a row are significantly different (*p* < 0.05).

## Data Availability

The data presented in this study are available upon request from the corresponding author.

## List of Abbreviations

NAFLD: non-alcoholic fatty liver disease; MS: metabolic syndrome; TG: triglycerides; NASH: non-alcoholic steatohepatitis; IR: insulin resistance; HCD-LFD: high carbohydrate, low-fat diet; HSF-LCD: high saturated fat, low carbohydrate diet; HMUSF: high monounsaturated fat diet; HMCF: high medium chains fat diet; HDHF: high carbohydrate, high-fiber diet; BW: body weight; H&E: hematoxylin and eosin; TC: total cholesterol; LDL-C: low-density lipoprotein cholesterol; HDL-C: high-density lipoprotein cholesterol; ALT: alanine aminotransferase; AST: aspartate aminotransferase; FE: food efficiency; HCDs: high carbohydrate diets; HFDs: high-fat diets; BMI: body mass index; VAT: visceral adipose tissue; SFA: saturated fatty acid; GIP: glucose-dependent insulinotropic polypeptide; FFA: free fatty acids; TNF-α: tumor necrosis factor alpha; GLP-1: glucagon-like peptide-1; EPA; eicosapentanoic acid; DHA; docosahexanoic acid; and HbA1c: Hemoglobin A1c.
